# Dual role of RACK1 in airway epithelial mesenchymal transition and apoptosis

**DOI:** 10.1111/jcmm.15061

**Published:** 2020-02-17

**Authors:** Yue Pu, Yuan‐qi Liu, Yan Zhou, Yi‐fan Qi, Shi‐ping Liao, Shi‐kun Miao, Li‐ming Zhou, Li‐hong Wan

**Affiliations:** ^1^ Department of Pharmacology West China School of Basic Medical Sciences & Forensic Medicine Sichuan University Chengdu Sichuan PR China; ^2^ Department of Intensive Care Unit West China Hospital Sichuan University Chengdu Sichuan PR China; ^3^ Grade 2015 West China School of Basic Medical Sciences & Forensic Medicine Sichuan University Chengdu Sichuan PR China; ^4^ Functional Laboratory West China School of Basic Medical Sciences & Forensic Medicine Sichuan University Chengdu Sichuan PR China

**Keywords:** apoptosis, epithelial mesenchymal transition (EMT), JNK/Smad3, RACK1, TGF‐β1

## Abstract

Airway epithelial apoptosis and epithelial mesenchymal transition (EMT) are two crucial components of asthma pathogenesis, concomitantly mediated by TGF‐β1. RACK1 is the downstream target gene of TGF‐β1 shown to enhancement in asthma mice in our previous study. Balb/c mice were sensitized twice and challenged with OVA every day for 7 days. Transformed human bronchial epithelial cells, BEAS‐2B cells were cultured and exposed to recombinant soluble human TGF‐β1 to induced apoptosis (30 ng/mL, 72 hours) and EMT (10 ng/mL, 48 hours) in vitro, respectively. siRNA and pharmacological inhibitors were used to evaluate the regulation of RACK1 protein in apoptosis and EMT. Western blotting analysis and immunostaining were used to detect the protein expressions in vivo and in vitro. Our data showed that RACK1 protein levels were significantly increased in OVA‐challenged mice, as well as TGF‐β1‐induced apoptosis and EMT of BEAS‐2B cells. Knockdown of RACK1 (siRACK1) significantly inhibited apoptosis and decreased TGF‐β1 up‐regulated EMT related protein levels (N‐cadherin and Snail) in vitro via suppression of JNK and Smad3 activation. Moreover, siSmad3 or siJNK impaired TGF‐β1‐induced N‐cadherin and Snail up‐regulation in vitro. Importantly, JNK gene silencing (siERK) also impaired the regulatory effect of TGF‐β1 on Smad3 activation. Our present data demonstrate that RACK1 is a concomitant regulator of TGF‐β1 induces airway apoptosis and EMT via JNK/Smad/Snail signalling axis. Our findings may provide a new insight into understanding the regulation mechanism of RACK1 in asthma pathogenesis.

## 1 INTRODUCTION

Asthma is a complicated airway inflammatory disease characterized by airway remodelling resulting from epithelial damage, glandular hyperplasia and subepithelial fibrosis.^1^ Current epidemiological results show that asthma has affected approximately 300 million individuals worldwide.^2^ A series of studies have demonstrated that airway epithelium is the first barrier against pathogens and allergens by the formation of the tight and adherens junctions to orchestrate airway inflammatory and airway remodelling responses.^3^ In asthmatic airways, repeated epithelial damage and repair triggers and activates airway remodelling. Thus, airway epithelium has been considered as a crucial therapeutic target in the progression of airway remodelling in respiratory allergic disease, especially asthma.^4^, ^5^ Unfortunately, none of the current therapy directly targets epithelial integrity or repair process.

Airway epithelial apoptosis and epithelial mesenchymal transition (EMT) are two important events that contribute to epithelial injury and repair process.^6^, ^7^, ^8^ Accumulating evidence has proved that loss of epithelial integrity caused by apoptosis allows airway and lung exposure to excess pathogens or environmental allergens,^9^ which further exacerbated airway epithelial apoptosis and impaired epithelial homoeostasis, and ultimately lead to airway remodelling and airway hyper‐responsiveness (AHR). On the other hand, to initiate tissue repair process, amounts of TGF‐β1 secreted from eosinophil and bronchial epithelial cell increase epithelial vulnerability to allergens and trigger the activation of EMT,^10^ which contributes to the subepithelial fibrosis in airway remodelling.^11^ Importantly, both of airway epithelial apoptosis and EMT are potently stimulated by TGF‐β, even simultaneously regulated by TGF‐β.^6^, ^12^ Thus, a deeper understanding of TGF‐β1‐induced airway epithelial apoptosis and EMT may provide a new therapeutic asthma treatment in epithelial injury‐repair process.

Receptor for activated protein kinase C 1 (RACK1), an integral component of ribosomes, is located at the basal airway epithelial cells^13^ that functions as the downstream target gene of TGF‐β1^14^ and plays an important role in multiple biological responses, including cell growth, differentiation, as well as migration.^15^ Our previous study demonstrated that OVA challenge significantly induced EMT process in airway epithelium via up‐regulation of TGF‐β1/RACK1,^16^ which is consistent with the report that knockdown RACK1 significantly inhibits the activation of TGF‐β/Smad signalling pathway in renal fibrosis.^17^ Moreover, it has been confirmed that RACK1 is required for regulating apoptosis although the role of RACK1 in regulating apoptosis remains controversial.^18^, ^19^, ^20^ However, the dual role and the potential mechanism of RACK1 in regulating airway epithelial apoptosis and EMT remain unclear.

To investigate the potential dual role of RACK1 in apoptosis and EMT of airway epithelial cells and the underlying mechanisms, OVA‐challenged mice and TGF‐β1‐induced apoptosis and EMT model in BEAS‐2B cells were used in this study. The present study showed RACK1 level was significant increased in OVA‐challenged mice and TGF‐β1‐induced apoptosis and EMT model in BEAS‐2B cells. Knockdown of RACK1 by siRNA markedly suppressed TGF‐β1‐induced apoptosis and EMT in BEAS‐2B cells via multiple signalling pathways, such as Smad3, JNK and ERK. Importantly, we found that and JNK/Smad3 pathway contributed to RACK1‐mediated airway EMT and epithelial apoptosis, while ERK pathway is mainly involved in regulating TGF‐β1‐induced EMT. The data presented suggest that RACK1 is a novel concomitant regulator in modulating airway epithelial apoptosis and EMT in response to TGF‐β1 stimulation via JNK/Smad3.

## 2 MATERIALS AND METHODS

### 2.1 Antibodies and inhibitors

Primary antibodies, second antibodies and inhibitors were shown in Table 1.

**Table 1:** Antibodies and inhibitors

Name	Experiment	Vendor	Dilution
Monoclonal anti‐RACK1 antibody	WB/IP/IF	Santa Cruz, CA	1:500
Polyclonal anti‐N‐cadherin antibody	WB	Abcam, UK	1:1000
Monoclonal anti‐JNK antibody	WB	Abcam, UK	1:1000
Monoclonal anti‐p‐JNK antibody	WB	CST, MA	1:1000
Monoclonal anti‐Smad3 antibody	WB/IP/IF	CST, MA	1:1000
Monoclonal anti‐p‐Smad3 antibody	WB	CST, MA	1:1000
Monoclonal anti‐E‐cadherin antibody	WB	CST, MA	1:1000
Monoclonal anti‐Snail antibody	WB	CST, MA	1:1000
Monoclonal anti‐Bcl‐2 antibody	WB	Abcam, UK	1:1000
Monoclonal anti‐Bax antibody	WB	Abcam, UK	1:1000
Polyclonal anti‐P53 antibody	WB	CST, MA	1:1000
Monoclonal anti‐GAPDH antibody	WB	ZSGB‐BIO, China	1:1000
Monoclonal anti‐β‐actin antibody	WB	ZSGB‐BIO, China	1:1000
Peroxidase‐conjugated goat anti‐rabbit IgG	WB	ZSGB‐BIO, China	1:2500
Peroxidase‐conjugated goat antimouse IgG	WB	ZSGB‐BIO, China	1:2500
SP600125 inhibitor		MCE, USA	
LY2157299 inhibitor		MCE, USA	

### 2.2 Animals

Male Balb/c mice weighing 20 ± 2 g (5‐6 weeks old) were purchased from Da‐Shuo Biological Technology Co., Ltd. All mice were housed at 22°C ± 1°C, humidity (60% ± 10%), on a 12 hours light/dark cycle with free access to food and water for 1 week. All animal experimental procedures were performed in accordance with the Committee on the Ethics of Animal Experiments of Sichuan University (Permit Number: 2003‐149). Animal ethics approval has been obtained from West China School of Basic Medical Sciences and Forensic Medicine.

### 2.3 Ova‐induced allergic airway inflammation mouse model

Twenty‐four mice were randomly divided into the control group (saline challenge, n = 12) and the OVA group (OVA challenge, n = 12). OVA‐induced allergic airway inflammation mouse model was performed as previously described.^16^ In brief, on days 0 and 14, mice in OVA group were sensitized with OVA (Sigma‐Aldrich, 20 μg) and aluminium hydroxide (Aldrich, 20 mg) in 0.2 mL of saline by intraperitoneal injection. Next, mice in OVA group were given aerosol challenges with 1%OVA for 60 minutes using an ultrasonic nebulizer (NE‐U12; Omron Co.) once daily for 7 consecutive days from day 21 to day 27. The mice in control group were challenged with saline instead of 1%OVA aerosol in a similar manner. Twenty‐four hours after the last challenge, all mice were killed. The inferior lobes of the right lungs (n = 6) were rapidly removed, dissected and stored at − 80°C for further Western blotting analysis. The left lung lobes were immediately fixed in 10% (v/v) neutral‐buffered formalin for 24 hours at 4°C and embedded in paraffin for immunofluorescence analysis.

### 2.4 Histopathological assessment

After fixed in 4% paraformaldehyde, the lung was embedded in paraffin and then cut into 4‐μm thick sections. The sections were stained with haematoxylin eosin (HE) and Masson trichrome staining (Masson) to assess the severity of lung pathology, leucocyte infiltration and degree of collagen deposition. The airway wall thickness (Wat), bronchial smooth muscle thickness (Wam), inflammatory degree and the degree of fibrosis were scored and quantified as our previously described.^16^, ^21^ All data and images were analysed blindly.

### 2.5 Cell culture and TGF‐β1 stimulation

Transformed human bronchial epithelial cells, BEAS‐2B cells were purchased from Shanghai Cell Bank (Shanghai, China) and cultured in Dulbecco's modified Eagle's medium (DMEM) (Harry Biotech) supplemented with 15% foetal bovine serum (FBS) (Gibco, 10,099‐141). Then, cells were seeded on 6‐well culture dishes at 1.5 × 10^5^ cells/mL. Experiments were performed when cells reached 70%‐80% confluence. Recombinant soluble human TGF‐β1 (Pepro Tech, 100‐21) was used to induced apoptosis (30 ng/mL, 72 hours) and EMT (10 ng/mL, 48 hours) in vitro, respectively. All experiments were conducted in triplicates and repeated three times.

### 2.6 Small interfering RNA (SIRNA) transfection

In BEAS‐2B cells, chemically synthesized, double‐stranded RACK1 siRNA (h, sc‐36354), double‐stranded Smad3 siRNA (h, sc‐38376) and negative control siRNA (NC, sc‐37007) were purchased from Santa Cruz. Double‐stranded JNK siRNA (h, 6232S) and double‐stranded ERK siRNA (h, 6560) were purchased from Cell Signalling Technology.

siRNA transfection was carried out using the lipofectamine3000 reagent (Invitrogen) according to the manufacturer's instructions. After incubation for 48 hours at 37°C, the transfected cells were treated with the absence (control) or presence of TGF‐β1. After 48 or 72 hours TGF‐β1 incubation, cells were harvested for further study. The knockdown efficiency was examined by Western blotting analysis. All experiments were conducted in triplicates and repeated three times.

### 2.7 Tunel staining and apoptotic rate (AI) measurement

The number of apoptotic cells in the lungs or BEAS‐2B cells were measured by the terminal deoxynucleotidyl transferase‐mediated dUTP nick end labelling (TUNEL) assay using TACS® 2 TdT diaminobenzidine kit (Trevigen, Gaithersburg, Md) to label the DNA damaged cells following the manufacturer's instructions. The AI was calculated in each lung of mice at 400 × magnification according to the following equation: AI = number of apoptotic cells/total number of nucleated cells × 100%. All data and images were analysed blindly.

### 2.8 Apoptosis assay by FCM

Apoptosis was measured using an annexin V‐fluorescein isothiocyanate (FITC) apoptosis detection kit (Annexin V‐FITC/PI, Vazyme Bio‐TECH, A211‐02). In brief, cells were plated at a density of 4 × 10^5^ cells per well into 6‐well plate and cultured in DMEM medium supplemented with 15% heat‐inactivated foetal bovine serum and antibiotics at 37°C in 5%CO_2_(v) in a humidified incubator for 24 hours and treated with presence of TGF‐β1 (30 ng/mL). Untreated cells were used as negative control. After 48 hours, the cells were harvested by trypsinization (Solarbio, T1350‐100), washed twice with cold PBS (2000 rpm, 5 minutes) and resuspended in 100 μL of binding buffer. Annexin V‐FITC (5 μL) and propidium iodide (PI, 5 μL) were added to each sample, and the mixture was incubated in the dark for 10 minutes at room temperature. Cells were immediately subjected to FACS analysis (BD Accuri C6) within 1 hour. Ex = 488 nm and Em = 530 nm. Both PI and Annexin V negative cells were considered as normal, PI negative and Annexin V positive cells were considered as early apoptotic, cells that were both PI and Annexin V positive were considered as late necrotic, and cells that were PI positive and Annexin V negative were considered as mechanically injured during the experiment. All the experiments were conducted in triplicates. Apoptosis rate (%) = number of apoptotic cells/total number of nucleated cells × 100%. All experiments were conducted in triplicates and repeated three times. All data and images were analysed blindly.

### 2.9 Protein preparation and western blotting analysis

The total proteins of lung or cells were extracted using RIPA Lysis Buffer (Beyotime Institute of Biotechnology, China) and the protein concentrations were measured with BCA protein assay kit (Beyotime Institute of Biotechnology, China). Equal amounts of total protein were loaded onto 10% SDS PAGE gels at 100 V for 60 minutes and electro transferred to PVDF membranes. After blocked in 5%no‐fat milk at room temperature for 2 hours, the membranes were incubated with the diluted antibodies at 4°C overnight. After washing with TBST, the membranes were probed with horseradish peroxidase‐conjugated secondary antibody at room temperature for 1 hour. Then proteins were detected by chemiluminescence reagent (GE Healthcare). Expression levels of the proteins were normalized by GAPDH or β‐actin as a loading control. Protein band density was quantified using Bio‐Rad Quantity One v4.62. All experiments were conducted in triplicates and repeated three times. All data and images were analysed blindly.

### 2.10 Immunofluorescence

Co‐staining of RACK1 and DAPI was performed on lung tissue sections as our previously described.^16^ In vitro, Beas‐2B cells were fixed, stained with primary antibodies against RACK1 (SC17754; 1:50; Santa Cruz, CA) overnight at 4°C and exposed to CY3 conjugated secondary goat antimouse IgG antibody (Invitrogen), counterstained with 4,6‐diamidino‐2‐phenylindole (DAPI) to visualize nuclei and analysed under a confocal microscope. All experiments were conducted in triplicates and repeated three times. All data and images were analysed blindly.

### 2.11 Statistics

Statistical analysis was performed by one‐way analysis of variance (ANOVA) with Bonferroni correction (GraphPad Prism version 4). All values were expressed as mean ± SEM, and *P* < .05 was considered statistically significant. Linear regression was applied to assess the correlation of two variables by chi‐squared test and the significance of difference was evaluated by ANOVA using SPSS software v.13.0.

## 3 RESULTS

### 3.1 Increases in RACK1 expression in OVA‐induced concomitant apoptosis and EMT

**Figure 1:**
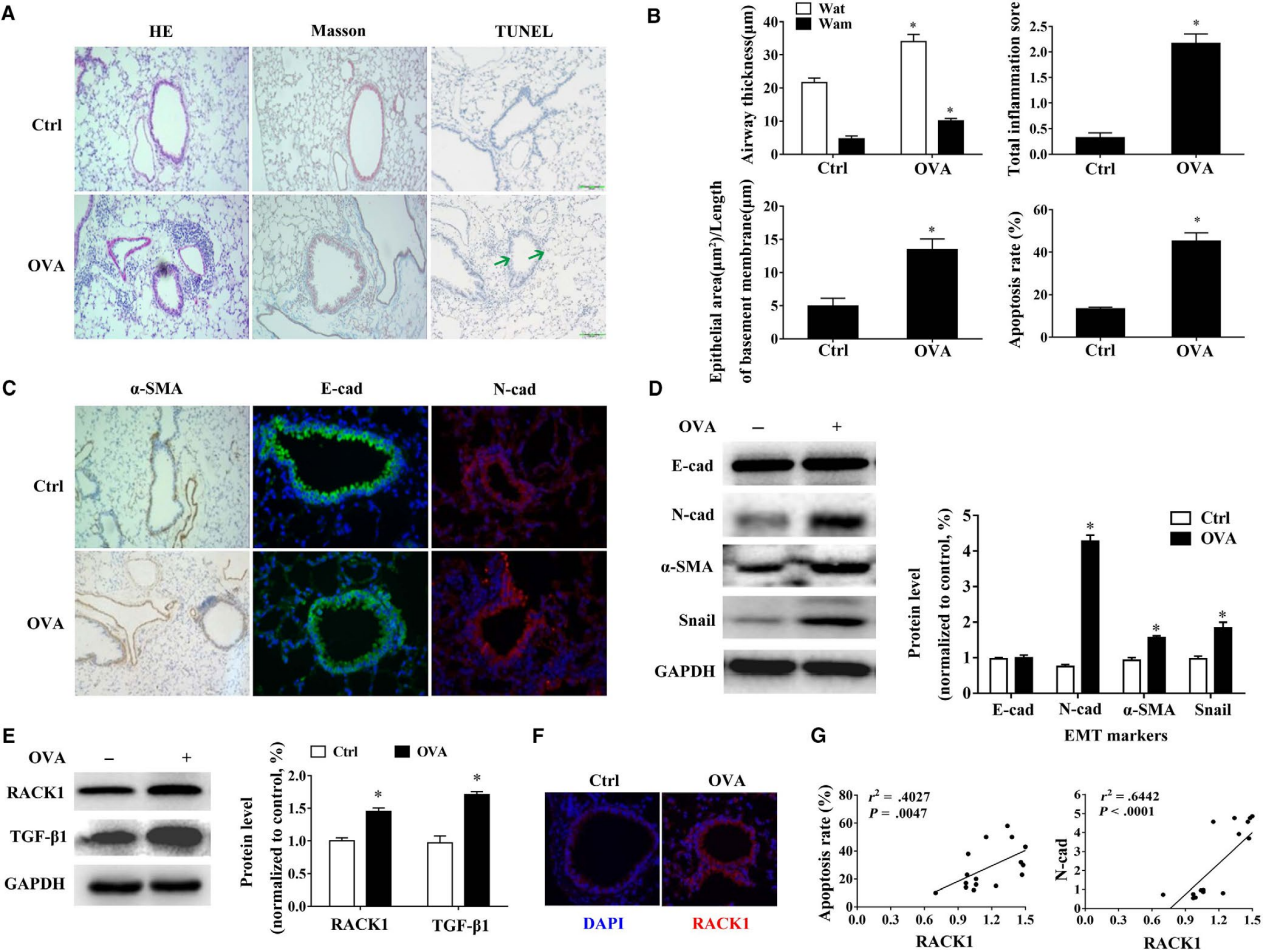
Increased RACK1 level in OVA‐induced concomitant apoptosis and EMT. OVA sensitization and challenge induce allergic airway inflammation mouse model. For histological analysis, lung tissue was collected at 24 h after the final OVA challenge. (A) Representative HE (magnification × 200), Masson (magnification × 200) and TUNEL stained sections of lung of mice in the control group and OVA group. Arrows indicated the TUNEL positive cells; (B) quantified results of airway wall thickness (Wat) and bronchial smooth muscle thickness (Wam), the total inflammation score, the degree of fibrosis and the apoptosis rate were analysed by Image‐Pro® Plus 6.0 software; (C) representative immunohistochemistry (IHC) (α‐SMA; magnification × 200) and immunofluorescent (E‐cadherin and N‐cadherin; magnification × 400)‐stained sections of lung of mice in the control group and OVA group; (D) Representative immunoblots (left panel) and quantitative analysis (right panel) of E‐cadherin, N‐cadherin, α‐SMA and Snail in lung of mice. GAPDH was used as an internal standard. The band intensity was analysed by Bio‐Rad Quantity One v4.62 software; (E) representative immunoblots (left panel) and quantitative analysis (right panel) of RACK1 and TGF‐β1 in lung of mice. GAPDH was used as an internal standard; (F) representative immunofluorescent of RACK1 in basal airway epithelial cells (red, magnification × 400); (G) positive correlation of expressions between RACK1 with apoptosis rate (*r* ^2^ = 0.403, *P* < .005) and RACK1 with N‐cadherin level (*r* ^2^ = 0.644, *P* < .0001). Values are presented as mean ± SEM ^*^ *P* < .05, vs control group (n = 6)

Ovalbumin (OVA) is the main mediator in allergic airway inflammation. In our study, mice upon OVA sensitization and challenge over a period of 7 days showed significant coughing, wheezing and dyspnoea, also characterized by an increased peribronchial infiltration of inflammatory cells, thickness of the peribronchial smooth muscle layer and collagen deposition (*P* < .05, Figure 1A), which were consistent with our previous study.^16^, ^21^ These results were confirmed again by the quantitative analysis as showed in Figure 1B. Moreover, a significant elevated amount of TUNEL^+^ cells were seen in the bronchial epithelium of OVA‐challenged mice (*P* < .05, Figure 1A) and the apoptosis rates were significantly higher in OVA‐challenged mice than those in the saline‐challenged mice (*P* < .05, Figure 1B). Alongside, OVA‐challenged mice demonstrated significant airway EMT, including the increase of expressions of mesenchymal markers (N‐cadherin, α‐SMA) and transcriptional factor Snail, and decrease of expression of epithelial markers (E‐cadherin) (*P* < .05, Figure 1C,D). However, Western blotting results showed a little difference between immunofluorescence (IF) and immunohistochemistry (IHC) results that there were no significant expressions difference in E‐cadherin levels between saline and OVA‐challenged mice (*P* < .05, Figure 1D). We thought the difference was as a result of the different sample source. These results indicated OVA sensitization and challenge induced concomitant apoptosis and EMT in allergic airway inflammation mice. Moreover, as we expected, TGF‐β1 protein levels in lung tissue were obviously up‐regulated in OVA‐challenged mice, as compared to saline‐challenged mice (*P* < .05, Figure 1E).

Subsequently, we examined the expression and localization of RACK1 in airway and found that RACK1 was mainly located in airway epithelial cells and notably up‐regulated in the lung tissue of OVA‐challenged mice (Figure 1E,F, *P* < .05), paralleled with elevation of TGF‐β1 protein level (Figure 1E, *P* < .05). Importantly, the correlation analysis revealed a significant positive correlation between RACK1 levels and apoptosis rate (*r* ^2^ = 0.403, *P* < .05, Figure 1G left panel) and RACK1 levels and N‐cadherin levels (*r* ^2^ = 0.644, *P* < .05, Figure 1G right panel), with higher RACK1 protein expression predicting a higher degree of apoptosis and EMT. Above results point towards the relationship between RACK1 levels and OVA‐induced concomitant apoptosis and EMT in allergic airway inflammation mice.

### 3.2 RACK1, a novel regulator in TGF‐β1‐induced apoptosis and EMT

**Figure 2:**
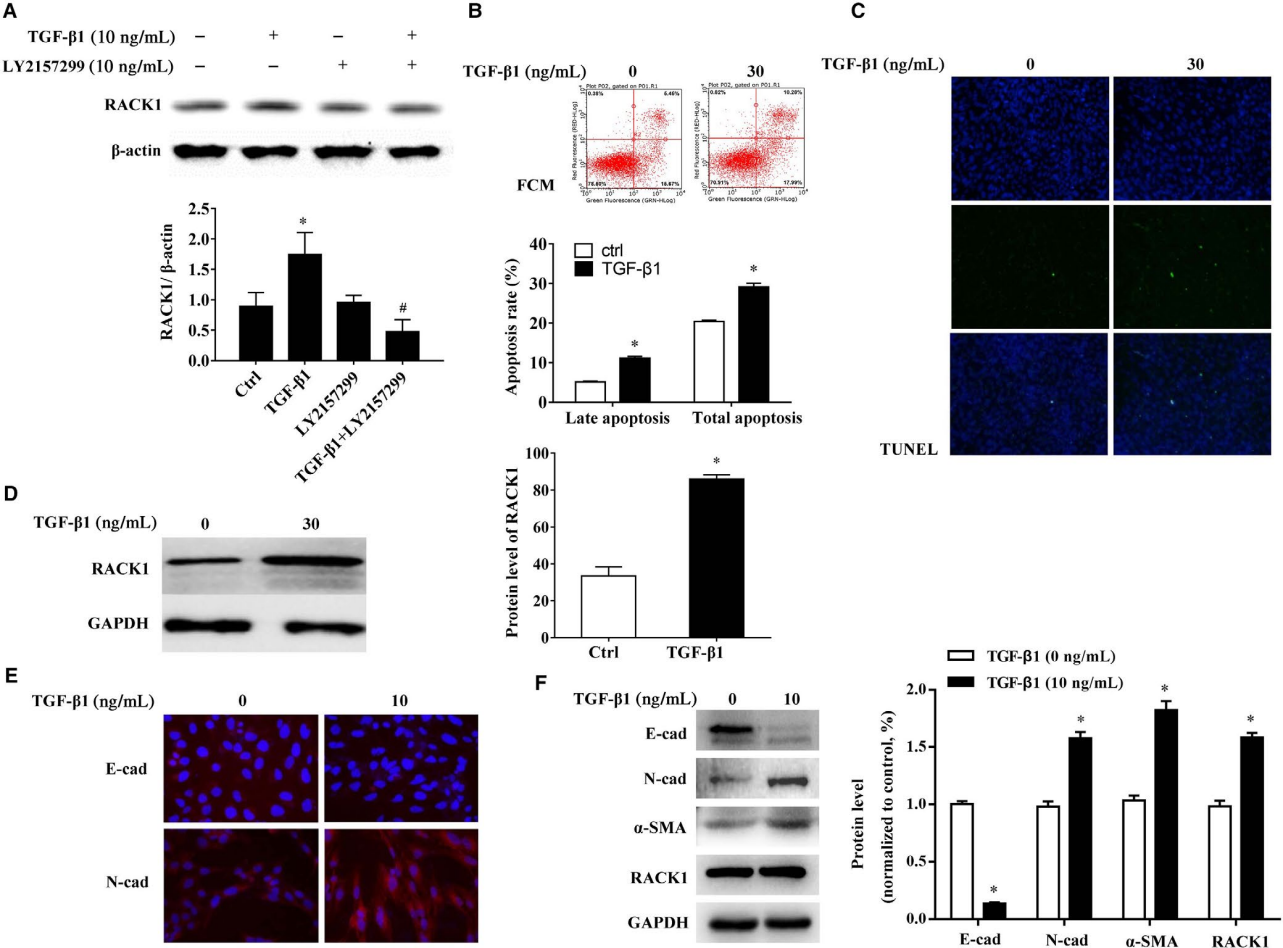
RACK1 up‐regulated in apoptosis and EMT of BEAS‐2B cells via TGF‐β1 pathway. (A) BEAS‐2B cells were exposed to recombinant human TGF‐β1 (10 ng/mL) and TGF‐β receptor I inhibitor (LY2157299, 10 μg/mL). Representative immunoblots (upper panel) and quantitative analysis (lower panel) of the relative protein level of RACK1 in cells. β‐actin was used as an internal standard. The band intensity was analysed by Bio‐Rad Quantity One v4.62 software; (B) BEAS‐2B cells were exposed to recombinant human TGF‐β1 (30 ng/mL, 72 h). Apoptosis in BEAS‐2B cells was demonstrated by flow cytometry (upper panel), the graph shows the apoptosis rate (lower panel); and (C) TUNEL stain; (D) representative immunoblots (left panel) and quantitative analysis (right panel) of the relative protein level of RACK1 in cells. GAPDH was used as an internal standard. The band intensity was analysed by Bio‐Rad Quantity One v4.62 software; (E) BEAS‐2B cells were exposed to recombinant human TGF‐β1 (10 ng/mL, 48 h). Representative immunofluorescent (E‐cadherin and N‐cadherin; magnification × 400) in cells; (F) representative immunoblots (left panel) and quantitative analysis (right panel) of E‐cadherin, N‐cadherin, α‐SMA and RACK1 in cells. GAPDH was used as an internal standard. The band intensity was analysed by Bio‐Rad Quantity One v4.62 software. Values are presented as mean ± SEM ^*^ *P* < .05, vs control group (n = 9)

To explore whether increase of RACK1 levels were mediated through the TGF‐β1 pathway, BEAS‐2B cells were exposed to recombinant human TGF‐β1 (10 ng/mL), TGF‐β receptor I inhibitor (LY2157299, 10 μg/mL), TGF‐β1 + LY2157299 or the culture medium (Ctrl) for 48 hours, respectively. Our results demonstrated that TGF‐β1 (10 ng/mL) significantly increased RACK1 levels as compared to Control (Figure 2A, *P* < .05). LY2157299 was highly effective in inhibiting TGF‐β1‐induced elevation of RACK1 (Figure 2A, *P* < .05).

Furthermore, as apoptosis and EMT can be potently stimulated by TGF‐β1,^6^, ^12^ to better understand the role of RACK1 in TGF‐β1‐induced apoptosis and EMT, recombinant soluble human TGF‐β1 was used to induced apoptosis (30 ng/mL, 72 hours) and EMT (10 ng/mL, 48 hours) in BEAS‐2B cells, respectively. Both of those doses were the best dose to induce apoptosis and EMT according to our preliminary experiments (data not shown) and publication.^12^, ^16^ As shown in Figure 2B,C, TGF‐β1 (30 ng/mL, 72 hours) significantly induced apoptosis of BEAS‐2B cells (*P* < .05). The ratio of late apoptosis and total apoptosis were significantly increased in the TGF‐β1 treated BEAS‐2B cells (*P* < .05). Moreover, Western blotting analysis showed that levels of RACK1 protein were significantly increased in TGF‐β1 treated BEAS‐2B cells compared with control cells (*P* < .05, Figure 2D), suggested that RACK1 is up‐regulated in BEAS‐2B cells during the development of apoptosis in vitro. Additionally, compared with control, TGF‐β1 (10 ng/mL, 48 hours) treatment significantly changed epithelial and mesenchymal marker protein levels (Figure 2E,F), such as decreased E‐cadherin expression and increased N‐cadherin and α‐SMA, as well as increased RACK1 level (Figure 2F, *P* < .05).

**Figure 3:**
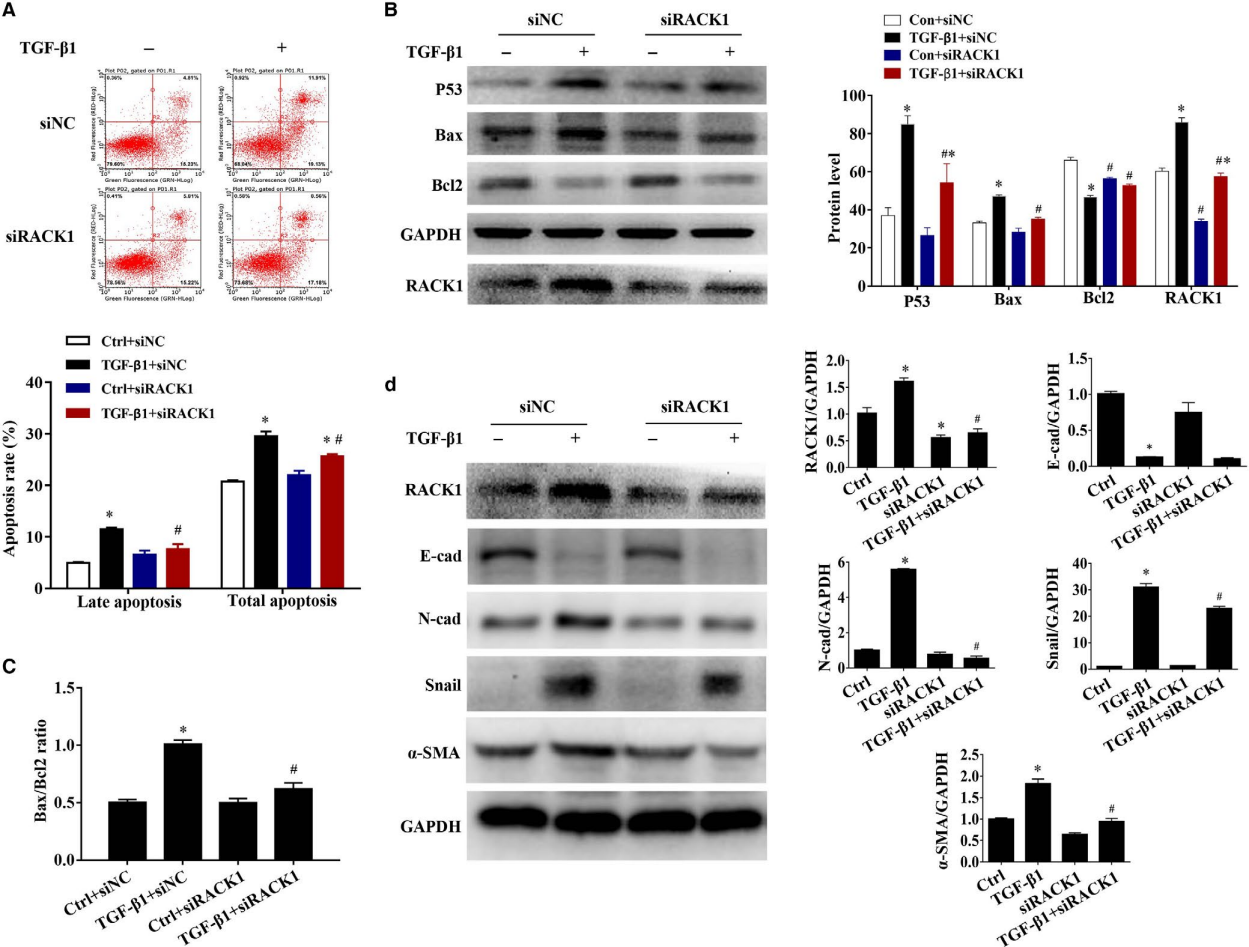
Knockdown of RACK1 inhibits TGF‐β1‐induced apoptosis and EMT of BEAS‐2B cells. BEAS‐2B cells were transfected with RACK1‐siRNA (siRACK1) or siRNA‐scramble (siNC) for 48 h and then exposed to recombinant human TGF‐β1 to induced apoptosis (30 ng/mL, 72 h) and EMT (10 ng/mL, 48 h). (A) Apoptosis in BEAS‐2B cells was demonstrated by flow cytometry (upper panel), and the graph shows the apoptosis rate (lower panel); (B) representative immunoblots (left panel) and quantitative analysis (right panel) of P53, Bax, Bcl2 and RACK1 in cells. GAPDH was used as an internal standard. The band intensity was analysed by Bio‐Rad Quantity One v4.62 software; (C) Bax/Bcl2 ratio; (D) representative immunoblots (left panel) and quantitative analysis (right panel) of E‐cadherin, N‐cadherin, α‐SMA, Snail and RACK1 in cells. GAPDH was used as an internal standard. The band intensity was analysed by Bio‐Rad Quantity One v4.62 software. Values are presented as mean ± SEM ^*^ *P* < .05, vs control group (n = 9). ^#^ *P* < .05, vs TGF‐β1 alone group (n = 9)

Subsequently, to elucidate the role of RACK1 in mediating TGF‐β1‐induced airway epithelial apoptosis and airway EMT, BEAS‐2B cells were transfected with RACK1‐siRNA (siRACK1) or siRNA‐scramble (siNC) to evaluate the effect of knockdown of RACK1 expression (siRACK1) on TGF‐β1‐induced apoptosis and EMT, respectively. As shown in Figure 3A, flow cytometric analysis indicated that knockdown of RACK1 expression (siRACK1) significantly inhibited TGF‐β1‐induced apoptosis in BEAS‐2B cells, especially late apoptosis (*P* < .05). Moreover, compared with siNC group, TGF‐β1‐induced increase of P53 and Bax expression and decrease of Bcl2 expression were significantly reversed by siRACK1 transfection (*P* < .05, Figure 3B). Especially, TGF‐β1‐induced increase of Bax/Bcl2 ratio was significantly inhibited in siRACK1 transfection group (*P* < .05, Figure 3C). TGF‐β1 induced the up‐regulation of RACK1 levels in BEAS‐2B cells (*P* < .05, Figure 3B), which were reversed by siRACK1 (*P* < .05) with the interference efficiency of 50%. Alongside, as expected, siRACK1 treatment could significantly alleviate the rise in RACK1 induced by TGF‐β1 in EMT model (Figure 3D, *P* < .05). We found that without TGF‐β1 exposure, siRACK1 treatment had no effect on the protein expressions of epithelial and mesenchymal markers (E‐cadherin, N‐cadherin, α‐SMA and Snail), suggesting that RACK1 did not induce EMT alone. However, knockdown of RACK1 significantly reversed elevation of mesenchymal markers (N‐cadherin, Snail and α‐SMA) in siRACK1‐treated cells in the presence of TGF‐β1 stimulant (Figure 3D, *P* < .05). Interesting, there was no significant effect on epithelial marker E‐cadherin (Figure 3D, *P* > .05). The inconsistent result between E‐cadherin and N‐cadherin may be because of the lower interfering efficiency of siRACK1 (~50%, Figure 3D), which is not enough to up‐regulate E‐ cadherin on this condition. These data suggest that RACK1 may be a novel regulator in TGF‐β1‐induced airway epithelial apoptosis and airway EMT.

### 3.3 RACK1‐mediated anti‐apoptotic and anti‐EMT function via multiple signalling pathways

**Figure 4:**
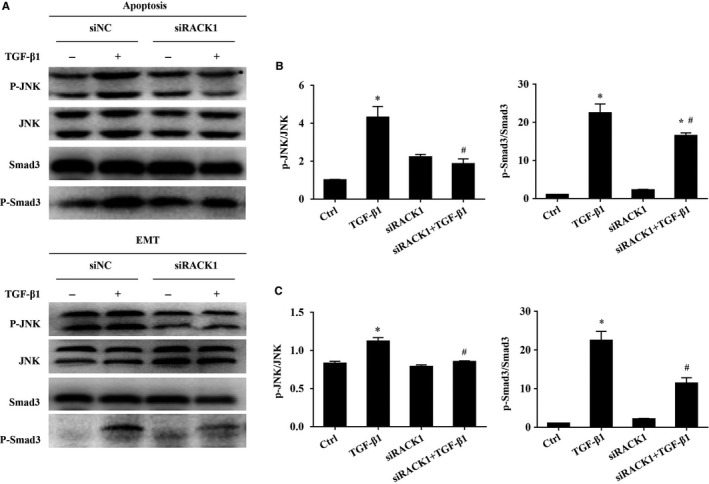
Knockdown of RACK1 inhibited JNK and Smad3 signal in TGF‐β1‐induced apoptosis and EMT. BEAS‐2B cells were transfected with RACK1‐siRNA (siRACK1) or siRNA‐scramble (siNC) for 48 h and then exposed to recombinant human TGF‐β1 to induced apoptosis (30 ng/mL, 72 h) and EMT (10 ng/mL, 48 h). (A) Representative immunoblots of p‐JNK, JNK, Smad3 and p‐Smad3 in TGF‐β1‐induced apoptosis of BEAS‐2B cell (upper panel) and TGF‐β1‐induced EMT of BEAS‐2B cell (Lower panel); (B) ratio of p‐JNK/JNK and p‐Smad3/Smad3 in apoptosis (C) and EMT. Values are presented as mean ± SEM ^*^ *P* < .05, vs control group (n = 9). ^#^ *P* < .05, vs TGF‐β1 alone group (n = 9)

It is well known that Smad‐independent signalling (MAPKs) is involved in the regulation of TGF‐β1‐induced apoptosis and EMT,^22^, ^23^, ^24^ as well as Smad‐dependent signalling. To further determine whether Smad‐dependent and Smad‐independent signalling (MAPKs) are associated with TGF‐β1‐induced apoptosis and EMT in BEAS‐2B cells, we quantified the expression levels of Smad3, p‐Smad3, JNK, p‐JNK, ERK and p‐ERK by Western blotting analysis. As shown in Figure S1A, p‐Smad3/Smad3, p‐JNK/JNK and p‐ERK/ERK in lung tissue were markedly enhanced in OVA‐challenged mice, compared with control mice (*P* < .05), indicating all the pathways were activated. Besides, a significant increase in Smad3, p‐JNK and p‐Smad3 protein levels were detected in TGF‐β1‐induced apoptosis of BEAS‐2B cells (Figure S1A, *P* < .05). But in TGF‐β1‐induced EMT of BEAS‐2B cells, following TGF‐β1 treatment, the total Smad3, JNK and ERK protein expressions were no significant difference compared with control cells (Figure S1A, *P* > .05). But the level of p‐Smad3, p‐JNK and p‐ERK were significantly increased in TGF‐β1‐stimulated cells (Figure S1A, *P* < .05), indicating Smad3, JNK and ERK were activated on the condition of EMT. As a result of our finding that the expression levels of ERK and p‐ERK were not altered in TGF‐β1‐induced apoptosis of BEAS‐2B cells (Figure S1A, *P* > .05), Smad3 and JNK pathways were selected to evaluate the regulatory effect of RACK1 in the subsequent experiments. As shown in Figure 4A, knockdown of RACK1 significantly suppressed Smad3 and JNK activation in TGF‐β1‐induced apoptosis (*P* < .05), but the ratio of p‐ERK/ERK was not significantly different after siRACK1 treatment compared to those of the vehicle control (Figure S2A, *P* < .05). Meanwhile, Smad3, JNK and ERK activation were significantly inhibited by knockdown of RACK1 (Figure 4A, Figure S2A, *P* < .05). To identify whether the RACK1 mediated TGF‐β1 induced EMT through interaction with Smad3, we co‐stained RACK1 and Smad3. Dual labelling immunofluorescence staining and confocal imaging analysis revealed that RACK1 was mostly co‐localized with Smad3 in cytoplasm of BEAS‐2B cells compared with control (Figure S2B), but Co‐IP analysis did not show any interaction directly between RACK1 and Smad3 (Figure S2C), indicating that RACK1 could regulate Smad3 activation indirectly.

**Figure 5:**
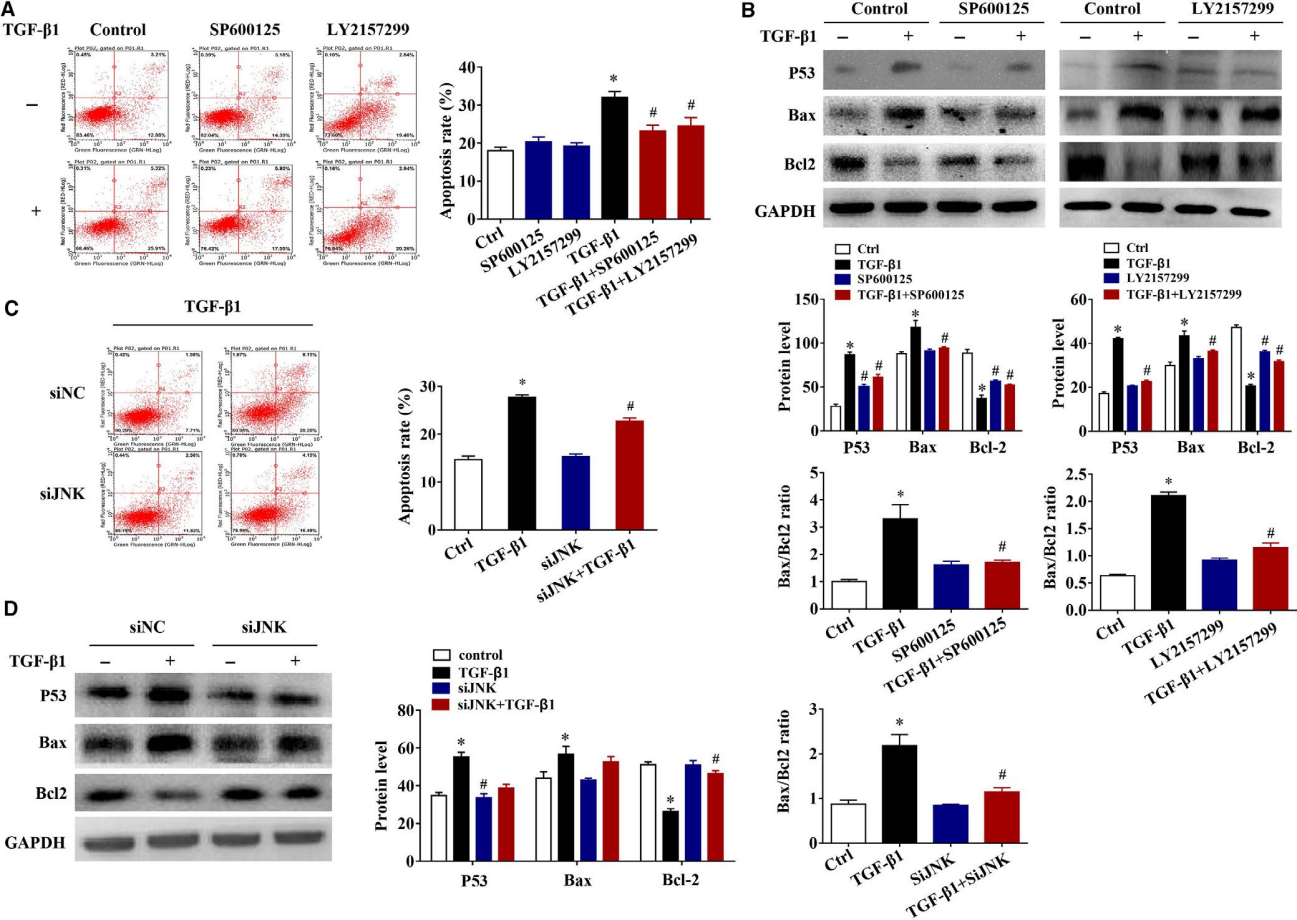
Role of JNK and Smad3 in TGF‐β1‐induced apoptosis of BEAS‐2B cells. (A) Effect of SP600125 (a JNK inhibitor) and LY2157299 (TGF‐β receptor I inhibitor) on apoptosis was demonstrated by flow cytometry (left panel), and the graph shows the apoptosis rate (right panel); (B) apoptotic relative proteins (P53, Bax and Bcl2) levels. GAPDH was used as an internal standard. The band intensity was analysed by Bio‐Rad Quantity One v4.62 software; (C) effect of siJNK on apoptosis was demonstrated by flow cytometry (left panel), and the graph shows the apoptosis rate (right panel); (D) effect of siJNK on apoptotic relative proteins (P53, Bax and Bcl2) levels. GAPDH was used as an internal standard. The band intensity was analysed by Bio‐Rad Quantity One v4.62 software. Values are presented as mean ± SEM ^*^ *P* < .05, vs control group (n = 9). ^#^ *P* < .05, vs TGF‐β1 alone group (n = 9)

**Figure 6:**
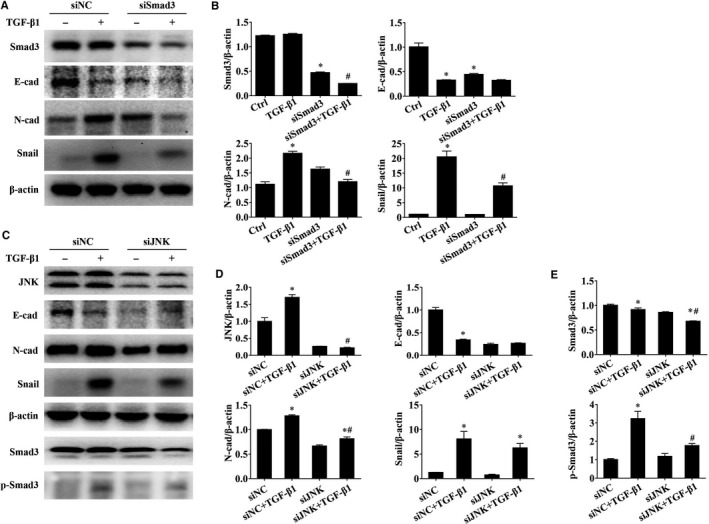
Role of JNK and Smad3 in TGF‐β1‐induced EMT of BEAS‐2B cells. BEAS‐2B cells were transfected with Smad3‐siRNA (siSmad3), JNK siRNA (siJNK) or siRNA‐scramble (siNC) for 48 h and then exposed to recombinant human TGF‐β1 (10 ng/mL, 48 h) to induced EMT. (A) Representative immunoblots and (B) quantitative analysis of the relative protein levels of Smad3, E‐cadherin, N‐cadherin and Snail in cells; (C) representative immunoblots and (D) quantitative analysis of the relative protein levels of JNK, E‐cadherin, N‐cadherin and Snail in cells; (E) quantitative analysis of the relative protein levels of Smad3 and p‐Smad3 in cells. β‐actin was used as an internal standard. The band intensity was analysed by Bio‐Rad Quantity One v4.62 software. Values are presented as mean ± SEM ^*^ *P* < .05, vs control group (n = 9). ^#^ *P* < .05, vs TGF‐β1 alone group (n = 9)

Next, to further characterize the Smad3 and JNK pathways in TGF‐β1‐induced apoptosis and EMT in BEAS‐2B cells and elucidate its importance, a series of kinase inhibitors, including SP600125 (a JNK inhibitor) and LY2157299 (a TGF‐β receptor I kinase inhibitor), and siRNA for knockdown of Smad3 and JNK were employed. As shown in Figure 5A,B, SP600125 (a JNK inhibitor) and LY2157299 (a TGF‐β receptor I kinase inhibitor) partially abrogated the TGF‐β1‐induced apoptosis and reversed TGF‐β1‐induced increase of P53 and Bax expressions and decrease of Bcl2 expression significantly (*P* < .05). The similar results were found after siJNK treatment in BEAS‐2B cells (Figure 5C,D, *P* < .05). Furthermore, we used the siSmad3 to knockdown Smad3 and observe the changes of EMT associated protein levels. According to the results of Western blotting analysis, knockdown of Smad*3* notably reduced the TGF‐β1‐induced elevation in the EMT mesenchymal marker N‐cadherin and Snail (Figure 6A,B, *P* < .05). Then, we used siJNK to knockdown JNK and observe the changes of EMT associated protein levels and expression and activation of Smad3. Based on the results of Western blotting analysis, knockdown of JNK significantly decreased the TGF‐β1‐induced elevation in the EMT marker N‐cadherin and Snail (Figure 6C,D, *P* < .05). Moreover, the total Smad3 and p‐Smad3 protein expressions were significantly inhibited by siJNK in the presence of TGF‐β1 (Figure 6E, *P* < .05), suggesting the potential role of JNK on Smad3 pathway in TGF‐β1‐induced EMT. However, consistent with those of siRACK1 treatment in Figure 3D, there was no significant effect on epithelial marker E‐cadherin after siJNK and siSmad3 treatment compared to those of the vehicle control (Figure 6A‐D, *P* > .05). The similar results were found after siERK treatment in TGF‐β1‐induced EMT of BEAS‐2B cells (Figure S3, *P* < .05). These data demonstrated that RACK1 mediated anti‐apoptotic and anti‐EMT function via multiple signalling pathways, including Smad‐dependent and Smad‐independent signalling (MAPKs). Among those, ERK pathway is mainly involved in regulating TGF‐β1‐induced EMT, but not TGF‐β1‐induced apoptosis.

## 4 DISCUSSION

Functioned as the downstream target gene of TGF‐β1,^14^ RACK1 has been implicated in the pathogenesis of fibrosis^17^ and apoptosis,^18^, ^19^, ^20^ but its involvement in the development of airway epithelial damage and repair remains largely unknown. In the present study, we have demonstrated an overexpression of RACK1 in a mouse model of allergic airway inflammation induced by OVA challenge and in TGF‐β1‐induced airway epithelial apoptosis and EMT model. Notably, knockdown of RACK1 by siRNA markedly inhibited TGF‐β1‐induced apoptosis and EMT in BEAS‐2B cells. Mechanistically, JNK/Smad3 signal activation concomitantly contributes to RACK1‐mediated airway epithelial apoptosis and EMT.

Accumulating evidence has suggested TGF‐β1‐related airway epithelial apoptosis process is the major morphological feature leading to epithelial cell injury.^21^, ^25^ Moreover, repeated airway epithelial cell damage and repair is a key cause of airway remodelling,^26^ because of enhancing activation of the epithelial mesenchymal trophic unit and leading to subepithelial airway fibrosis.^27^ Notably, some recent studies indicate that as a potent pleiotropic cytokine, TGF‐β1 even simultaneously induces the apoptosis and EMT in certain epithelial cells, such as renal tubular epithelial cells,^28^ mammary epithelial cells^12^ and lens epithelial cells.^29^ In this study, we have provided substantial evidence for the first time to support the concomitant airway epithelial apoptosis and EMT in OVA‐challenged mice, as indicated by apoptosis index and the induction of four well known EMT markers: E‐cadherin, N‐cadherin, α‐SMA and Snail. Moreover, in the lungs of OVA‐challenged mice, we observed higher protein expressions of TGF‐β1 and RACK1, the downstream target gene of TGF‐β1,^14^ in comparison with control mice. Interestingly, RACK1 has been identified as a multifunctional anchoring or adaptor protein in regulating cell growth, differentiation and migration^15^ and is potentially associated with asthma.^17^ In this study, consistent with previous reports,^13^ we found RACK1 was located at the airway epithelial cells. In addition, the expressions of RACK1 protein were significant increased in the lungs of OVA‐challenged mice and positive correlated with apoptosis rate and N‐cadherin (an important EMT marker). Hence, up‐regulation of RACK1 may mediate the concomitant TGF‐β1‐induced apoptosis and EMT by in OVA‐challenged mice.

Additionally, RACK1 has been found to act as a scaffold protein for PKC and interact with a variety of proteins in the regulation of apoptosis^18^, ^19^, ^20^ and fibrosis.^17^ To explain the relationship between RACK1 and TGF‐β1‐induced apoptosis and EMT process, we performed a mechanistic study to determine whether inhibition of RACK1 expression significantly attenuated TGF‐β1‐induced apoptosis and EMT in airway epithelial cells. TGF‐β1 has a marked induction effect in both apoptosis^30^ and EMT^31^ in airway epithelial cells. Our current study used 30 ng/mL TGF‐β1 stimulated cells for 72 hours and 10 ng/mL TGF‐β1 stimulated cells for 48 hours that induced apoptosis and EMT according to our previous work, both of which was the best dose to induce apoptosis and EMT, respectively. Our findings indicated that in TGF‐β1‐induced apoptosis and EMT of BEAS‐2B cells, RACK1 levels were markedly up‐regulated, too. Moreover, knockdown RACK1 significantly suppressed TGF‐β1‐induced apoptosis and EMT in BEAS‐2B cells, which implied that the contribution of RACK1 is important in regulating TGF‐β1‐induced airway epithelial apoptosis and EMT. Further, significant decrease of P53 and Bax expression and increase of Bcl2 expression after RACK1 knockdown in TGF‐β1‐induced airway epithelial apoptosis indicates a crucial role of RACK1 in the regulation of TGF‐β1‐related airway epithelial injury. Similarly, significant decrease of three mesenchymal markers, such as N‐cadherin, Snail and α‐SMA after RACK1 knockdown in TGF‐β1‐induced airway EMT also indicates a crucial role of RACK1 in the regulation of TGF‐β1‐related airway epithelial repair process.

c‐Jun N‐terminal kinase (JNK), belongs to the superfamily of MAP‐kinases, is known to be recruited to ribosomes and activated by RACK1.^32^, ^33^ It is well known that the phosphorylated JNK translocates to nucleus to promote apoptosis by activation of p53 and increase of the expression of several pro‐apoptotic genes such as Bax.^34^, ^35^, ^36^ In the present study, JNK was significantly activated in TGF‐β1‐induced apoptosis of BEAS‐2B cells, while JNK inhibitor SP600125 or knockdown of JNK significantly suppressed the expression of pro‐apoptotic genes P53 and Bax and increased the expression of Bcl2. These results were consistent with prior report that inhibition or silence of JNK inhibited p53‐dependent apoptosis in response to DNA‐damage.^35^ Importantly, our data demonstrated that RACK1 gene silencing (siRACK1) impaired the regulatory effect of TGF‐β1 on activation of JNK in apoptosis of BEAS‐2B cells, suggesting the involvement of RACK1/JNK in airway epithelial apoptosis under this condition. On the other hand, as one of the best‐characterized non‐Smad pathway utilized by TGF‐β, JNK cascade play a predominant role in the pathological process of TGF‐β1‐induced EMT^37^ through activation of Smad3 pathway,^38^ which is one of the main downstream signal molecules of TGF‐β1 receptors. Our subsequent results revealed that JNK was significantly activated in TGF‐β1‐induced EMT of BEAS‐2B cells, while knockdown of JNK significantly decreased the TGF‐β1‐induced elevation of N‐cadherin and Snail, as well as Smad3 protein expression and activation.

Similarly, knockdown RACK1 significantly impaired the regulatory effect of TGF‐β1 on activation of JNK and Smad3 in TGF‐β1‐induced apoptosis and EMT in BEAS‐2B cells, suggesting JNK/Smad3 pathway contributed to RACK1‐mediated airway EMT and epithelial apoptosis. Obviously, above results support that JNK signal can promote cell death and fibrosis^38^ and highlight the potential dual role of RACK1 on regulating TGF‐β1‐induced airway epithelial apoptosis and EMT via JNK/Smad3 signal.

There are limitations in our study in respect to cell types and animal model. Primary human bronchial cells from asthmatic patients and conditioned knockout mice would have provided further insight into the beneficial effect of RACK1 in asthma.

**Figure 7:**
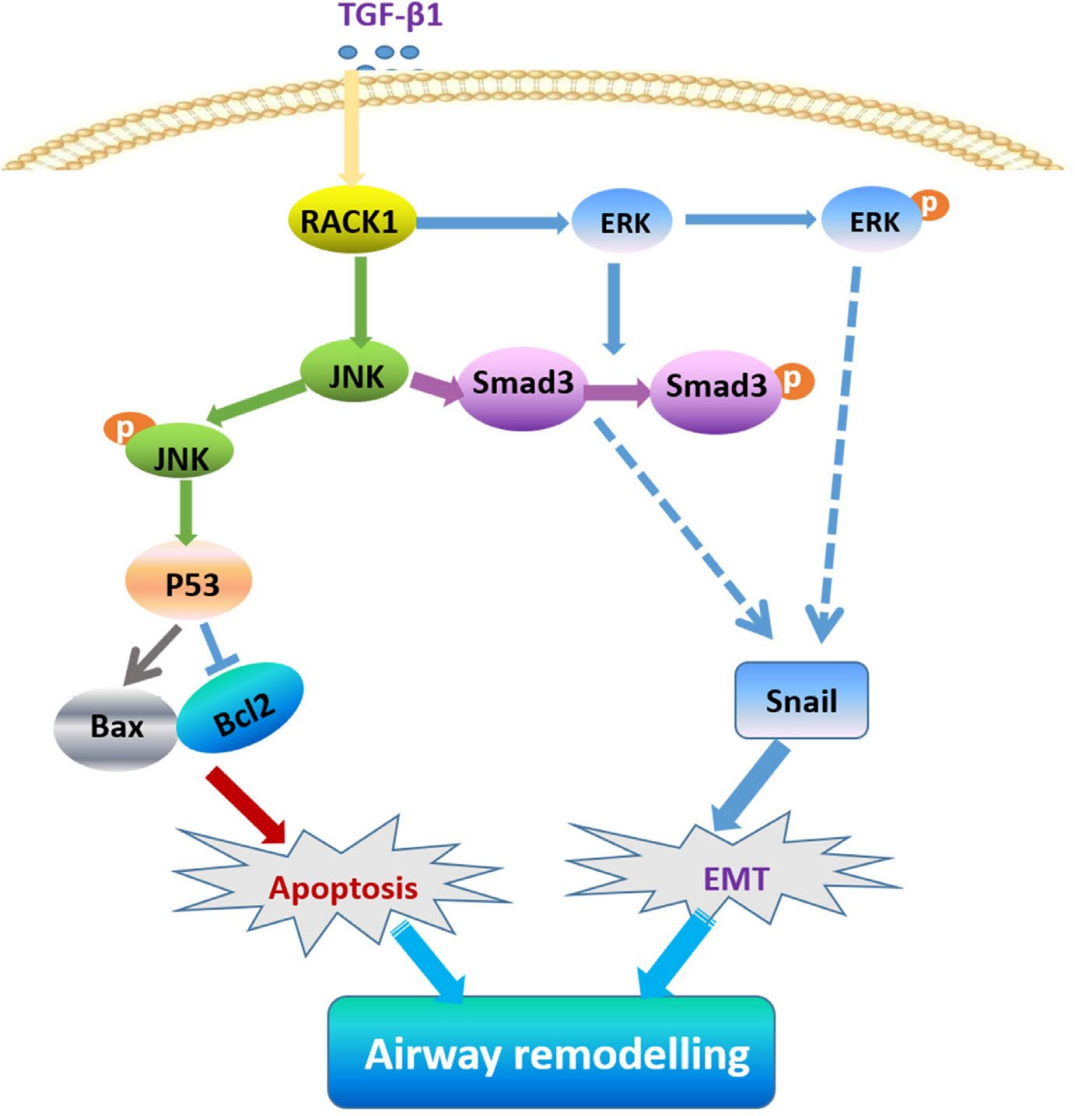
Schematic model of the proposed cellular mechanism. RACK1 is a novel concomitant regulator in modulating airway epithelial apoptosis and EMT in response to TGF‐β1 stimulation. Moreover, multiple signaling pathways contribute to RACK1‐mediated anti‐apoptotic and anti‐EMT function, including Smad3 and MAPKs

In conclusion, we have demonstrated that RACK1 is a novel concomitant regulator in modulating airway epithelial apoptosis and EMT in response to TGF‐β1 stimulation. Multiple signalling pathways contribute to RACK1‐mediated anti‐apoptotic and anti‐EMT function, including Smad3 and MAPKs (Figure 7).

## CONFLICT OF INTERESTS

The authors declare no conflict of interest.

## AUTHORS’ CONTRIBUTIONS

Li‐hong Wan conceived the idea, designed the study and wrote the manuscript. Yue Pu, Yuan‐qi Liu, Yan Zhou and Yi‐fan Qi conducted most of the cell and molecular biology experiments. Shi‐ping Liao and Shi‐kun Miao conducted animal experiment. Li‐ming Zhou revised the manuscript. All authors contributed to data analysis and the preparation, and final approval of the manuscript.

## Data Availability

The data that support the findings of this study are available on request from the corresponding author. The data are not publicly available due to privacy or ethical restrictions.
