# Supplementary material for: Dual role of RACK1 in airway epithelial mesenchymal transition and apoptosis
Source: J Cell Mol Med. 2020 Feb 17;24(6):3656–68. doi: 10.1111/jcmm.15061 (PMC7131927; doi:10.1111/jcmm.15061)
Supplement: Supplementary file 1 [file JCMM-24-3656-s001.pdf]

Supplemental Material and Methods

Co-localization of RACK1 and Smad3

Localization of RACK1 or Smad3 was examined by immunofluorescence staining.

Lung sections were incubated overnight at 4°C with primary antibodies raised against RACK1 (SC17754; 1:100; Santa Cruz, CA) and CY3 conjugated goat anti-mouse IgG antibody (GB25301; 1:300; Guge, China), then incubated with the sections for 50 min at room temperature. Nucleus was counterstained with 4,6-diamidino-2-phenylindole (DAPI). Images were captured using an Olympus microscope (IX51; Olympus Corporation, Tokyo, Japan).

BEAS-2B cells were fixed with 4% paraformaldehyde in PBS at room temperature for 0.5 h, and then incubated with primary antibodies against RACK1 (SC17754; 1:50; Santa Cruz, CA) and Smad3 (9523; 1:100; CST, MA) overnight at 4°C, followed by CY3 conjugated secondary goat anti-mouse IgG antibody or Alexa Fluor-488-conjugated secondary goat anti-mouse antibody (Invitrogen) at the room temperature. Nucleus was counterstained with 4,6-diamidino-2-phenylindole (DAPI). The cells were examined using a Leica AOBSTM SP2 confocal microscope (Leica Microsystems GmbH, Heidelberg, Germany) and data were processed via Image Pro Plus.

Co-immunoprecipitation (co-IP) assays

Immunoprecipitation was carried out to assess the interaction between RACK1 and Smad3. After harvesting the BEAS-2B cells, total protein extracts were centrifuged at 4°C with 12,000 g for 15 min. 300 μl of supernatant was collected and incubated with 5 μg of corresponding antibodies or IgG as control and 50 μl protein A/G magnetic beads (Biotool, USA) to immunoprecipitate RACK1 at 4°C. The immunocomplexes were then washed three times with lysis buffer. After performing magnetic separation and discarding the supernatant, pellets were boiled with 1xSDS loading buffer at 100°C for 10 min and then processed by western blot analysis.

Supplemental Figures and legends

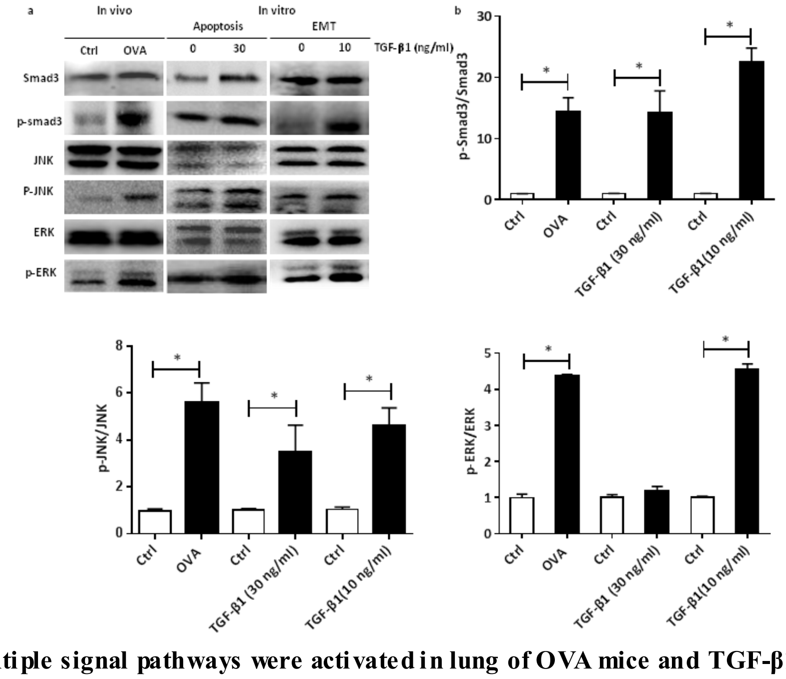

Figure S1 Multiple signal pathways were activated in lung of OVA mice and TGF-β1-induced apoptosis and EMT of BEAS-2B cells.

(a) Representative immunoblots of Smad3 and p-Smad3, JNK, p-JNK, ERK and p-ERK; (b) Quantitative analysis of Smad3 and p-Smad3, JNK, p-JNK, ERK and p-ERK. The band intensity was analyzed by Bio-Rad Quantity One v4.62 software. Values are presented as mean ± S.E.M. *P<0.05, versus control group (n=9).

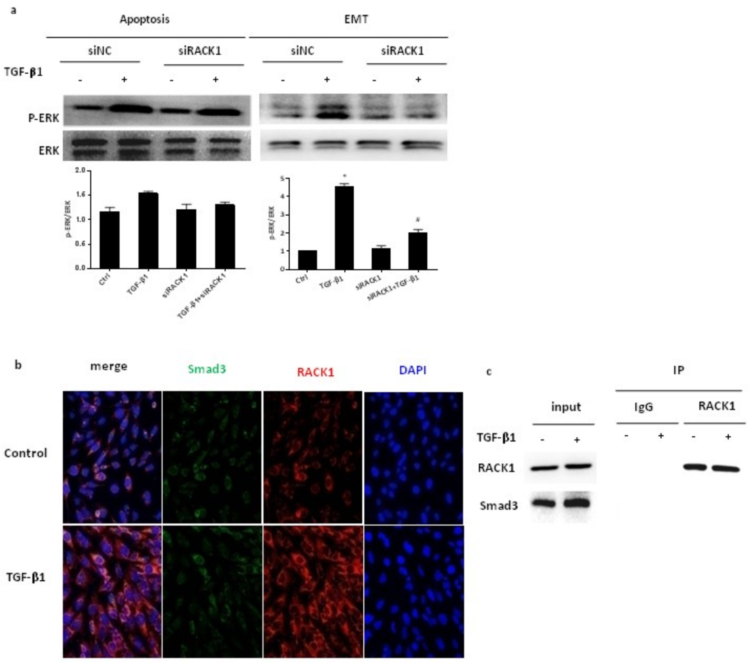

Figure S2 Effect of RACK1 on ERK and Smad3.

(a) BEAS-2B cells were transfected with RACK1-siRNA (siRACK1) or siRNA-scramble (siNC) for 48 h and then exposed to recombinant human TGF-β1 to induced apoptosis (30 ng/ml, 72 h) and EMT (10 ng/ml, 48 h). Representative immunoblots (upper panel) and quantitative analysis of ERK and p-ERK (lower panel). The band intensity was analyzed by Bio-Rad Quantity One v4.62 software. Values are presented as mean ± S.E.M. *P<0.05, versus control group (n=9); (b) Immunofluorescence indicated the location of RACK1 (Red), Smad3 (Green) in BEAS-2B cells. (c) Expressions and CoIP of RACK1 and Smad3. The RACK1 protein was immunoprecipitated with anti-RACK1 antibody, and the presence of Smad3 protein was detected by immunoblot analysis with anti-Smad3 antibody. These experiments were repeated three times.

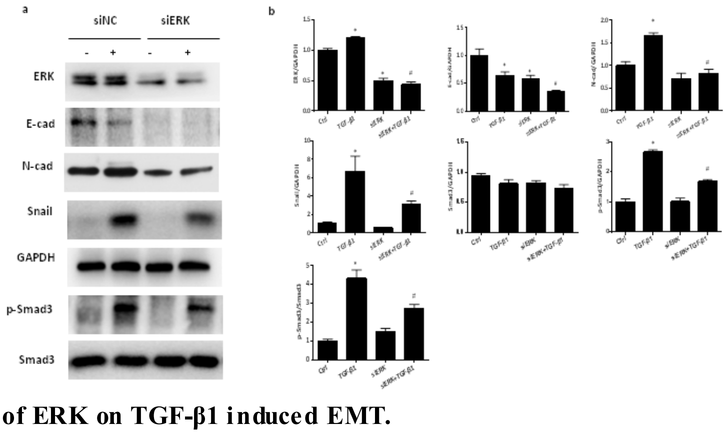

Figure S3 Effect of ERK on TGF-β1 induced EMT.

BEAS-2B cells were transfected with ERK-siRNA (siERK) or siRNA-scramble (siNC) for 48 h and then exposed to recombinant human TGF-β1 (10 ng/ml, 48 h) to induce EMT. (a) Representative immunoblots and (b) quantitative analysis of ERK, E-cadherin, N-cadherin, Snail, p-Smad3 and Smad3 levels in cells. The band intensity was analyzed by Bio-Rad Quantity One v4.62 software. Values are presented as mean ± S.E.M. *P<0.05, versus control group (n=9). #P<0.05, versus TGF-β1 alone group (n=9).
